# Population connectivity and larval dispersal of the exploited mangrove crab *Ucides cordatus* along the Brazilian coast

**DOI:** 10.7717/peerj.4702

**Published:** 2018-04-30

**Authors:** Fábio B. Britto, Anders J. Schmidt, Adriana M.F. Carvalho, Carolina C.M.P. Vasconcelos, Antonia M. Farias, Paul Bentzen, Fábio M. Diniz

**Affiliations:** 1 Department of Biology, Universidade Federal do Piauí, Teresina, Piauí, Brazil; 2 Universidade Federal do Sul da Bahia, Teixeira de Freitas, Bahia, Brazil; 3 Molecular Biology & Biotechnology Laboratory, Embrapa Meio-Norte, Teresina, Piauí, Brazil; 4 Universidade Federal do Piauí, Northeast Biotechnology Network RENORBIO, Teresina, Piauí, Brazil; 5 Department of Biology, Dalhousie University, Halifax, NS, Canada; 6 Molecular Biology Laboratory, Embrapa Caprinos e Ovinos, Sobral, Ceará, Brazil

**Keywords:** Population structure, Microsatellites, Gene flow, Conservation, Migration rate

## Abstract

**Background:**

The mangrove crab *Ucides cordatus* is considered a key species for the ecological balance of mangrove forests and a major source of employment and income for traditional crab collectors in Brazil. Several studies evidenced weak genetic variation among populations due to an efficient larval transport. However, gene flow patterns of the species is poorly understood, with no information about migration rates. The influence of the two main Brazilian currents in larval dispersion is also not clear. In order to provide baseline information for conservation, planning and management of this important fishery resource, the present study aimed to estimate and evaluate spatial distribution of genetic diversity, migration rates and gene flow directivity among populations of *U. cordatus* in Brazil.

**Methods:**

Nine microsatellites were used to resolve population structure of 319 crabs collected from six sites located along the Brazilian coast. The degree of geographical differentiation included estimates of genetic diversity, population structure and gene flow models, with spatial analysis of shared alleles (SAShA), isolation by distance tests, AMOVA, discriminant analysis of principal components (DAPC) and Bayesian clustering. We estimated the amount of ongoing gene flow between clusters using the coalescent-based method implemented in Migrate-N.

**Results:**

Loci were highly polymorphic (average of 12.4 alleles per locus) evidencing high genetic variability. There was significant differentiation among localities, despite of the low value of *F*_ST_ (= 0.019; *P < *0.001). *F*_ST_ and Jost’s *D* indexes were also estimated in pairwise comparisons and showed significant differences between most of the surveyed site pairs (*P <* 0.05). Structure evidenced a single genetic group among samples, however SAShA pointed to a non-panmictic condition (*P = *0.011). AMOVA detected four statistical significant clusters with low level of differentiation (*F*_CT_ = 0.037; *P = *0.023). The gene flow model that best described the population connectivity was the island model, with ∼24 crabs being exchanged among localities per generation.

**Discussion:**

The high migration rates found among localities seem to be the main force acting to sustain the distribution of the genetic diversity of *U. cordatus*. Despite the high gene flow and the weak population structure among samples, the significant genetic differences found suggest that gene flow alone does not bypass the effects of genetic drift, natural selection and/or human exploitation. These findings are vital for the establishment of a database to be used in the development of conservation programs.

## Introduction

The mangrove crab *Ucides cordatus* is one of the most typical inhabitants of mangrove ecosystems in Central and South Americas (Melo, 1996). It is abundant along Brazilian coast, where crab fishing is one of the most important activities in several estuaries, and a source of employment and income for many traditional crab collectors (Diele, Koch & Saint-Paul, 2005). This decapod crustacean is also considered a key species for the ecological balance within benthic communities of mangrove forests, and have the largest biomass among mangrove invertebrates (Wolff, Koch & Isaac, 2000). The species has a strong impact on litter turnover, nutrients cycling, soil aeration and forest productivity (Pülmanns et al., 2014, 2016). Despite its importance to the ecosystem and abundance throughout the year, *U. cordatus* is currently on the Brazilian list of species threatened by overfishing (Pinheiro & Rodrigues, 2011). In addition to exploitation and habitat destruction, a fungal disease has affected crab populations in several locations (Boeger et al., 2005, 2007; Schmidt, Theil & Galli, 2008; Vicente et al., 2012). Together, these events could reduce genetic diversity and eliminate well-adapted stocks, which would cause the species to decline (Avise & Hamrick, 1996).

This crab belongs to the infra-order Brachyura, family Ucididae (Crustacea: Decapoda) and has been extensively studied in Brazil. Its high fecundity are likely to cause direct impact on the species ecology and conservation. Recorded values of fecundity per female ranged from 36.081 to 250.566 eggs (Pinheiro, Baveloni & Leme-Terceiro, 2003) while fertility ranged from 71.200 to 220.800 larvae (Hattori & Pinheiro, 2003). Also, the efficient larval dispersion is important to sustain high levels of genetic diversity. The process begin with crabs performing a dramatic synchronized mating movements that allow larval emission specifically on the strongest syzygy ebb tide of the month (around new moon or full moon), following the “syzygy tide inequality cycle,” which provides efficient larval transport to ocean waters (Schmidt, Bemvenuti & Diele, 2012). The euryhaline planktonic *U. cordatus* zoea I and II larvae are able to tolerate a wide range of salinities. However, after these early larval phases, which take about eight days, the final development stages do not tolerate low salinities. Therefore, export of newly hatched larvae is an important strategy to avoid stressful salinities (Diele & Simith, 2006; Simith & Diele, 2008). Studies have also shown that the development until megalopal stage lasts 20–69 days, depending on the temperature and salinity conditions (Rodrigues & Hebling, 1989; Abrunhosa, Melo & Abrunhosa, 2003; Diele & Simith, 2006), which is enough time for long range dispersion. The plankton-to-benthos transition begins with the reinvasion of the estuary by the last larval stage—the megalopa. Megalopae settlement and metamorphosis occurs mainly at conspecific burrows (Schmidt & Diele, 2009), triggered by odors emanated by juveniles and adults (Simith, Abrunhosa & Diele, 2013). Adults reach the legal minimum capture size after 6.13 years in males and 7.38 years in females (Diele & Koch, 2010).

Several studies have reported high genetic diversity for the *U. cordatus* using molecular markers as Random Amplified Polymorphic DNA (RAPD), Polymerase Chain Reaction-Restriction Fragment Length Polymorphism (PCR-RFLP) (Oliveira-Neto et al., 2007a), control region mtDNA sequences (Oliveira-Neto et al., 2007b; Pie et al., 2008), and Inter-Simple Sequence Repeats (ISSR) (Britto et al., 2011). These studies have also not provided any evidence of population structure among surveyed sites. However, in widespread species, microsatellite markers present better resolution to detect any trace of genetic differentiation among populations that live apart, and may be under unequal forces of natural selection. Usually these markers have exceptional degree of polymorphism and informativeness for evolutionary and ecological studies at large or even fine scale surveys (Abdul-Muneer, 2014). More recently, a research using these markers in *U. cordatus* (Oliveira-Neto et al., 2014) evidenced weak but significant genetic differences among populations (*F*_ST_ = 0.03, *P* = 0.0014). Even though, no quantitative information on the gene flow magnitude or its possible directivity were evaluated until now. Bayesian analysis with Markov chain Monte Carlo methods can be used to provide reliable gene flow estimates without the unrealistic assumption of symmetry of migration rates or equal population sizes (Faubert, Waples & Gaggliotti, 2007). A method proposed by Beerli & Felsenstein (2001) uses molecular data to determine the sample genealogy and provide migration estimates even in cases where the differentiation among populations is assumed insignificant. Besides, different models regarding migration direction can be tested to detect possible routs of larval dispersion along the ocean.

**Figure 1:**
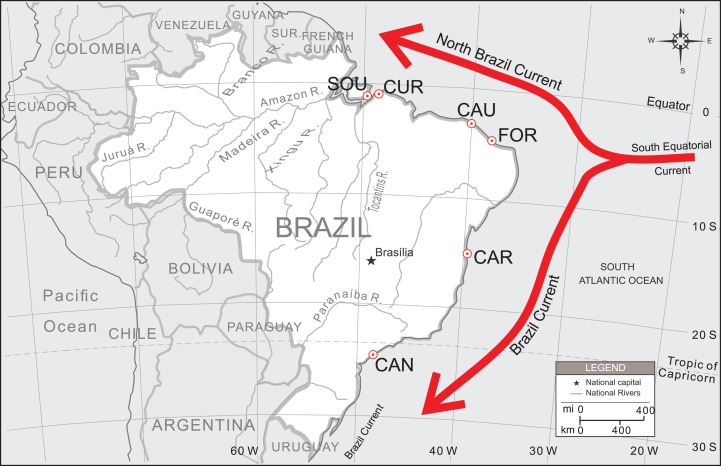
Sampling localities of *U. cordatus* populations surveyed along the Brazilian coast. FLO, Florianópolis—SC (*n* = 11); CAN, Cananéia—SP (*n* = 100); CAR, Caravelas—BA (*n* = 80); FOR, Fortim—CE (*n* = 54); GOI, Goiabeiras—CE (*n* = 23); CAU, Caucaia—CE (*n* = 48); PAR, Parnaíba—PI (*n* = 26); CUR, Curuçá—PA (*n* = 50); and SOU, Soure—PA (*n* = 50). The directions of the North Brazil Current (NBC) and the Brazil Current (BC) are also shown.

Due to the high ecological and social value of this species, a more extensive survey coverage also needs to be undertaken, particularly in estuaries under the possible influence of the North Brazil Current (NBC) (Fig. 1). Oliveira-Neto et al. (2014) concentrated most of their sample (77%) along a small portion of the Brazilian northeastern coast (over approx. 370 km), with only two collecting sites located on its lower and upper distribution limits. In this article we covered other sampling localities and used other set of microsatellites markers aiming to: (i) assess the spatial distribution of *U. cordatus* genetic diversity along the Brazilian coastline, including estuaries under the influence of the NBC and Brazil Current (BC), two major oceanic circulation systems flowing in opposite directions, away from each other; (ii) test different models of migration to estimate larval dispersal directivity; and (iii) estimate the migration rates among localities. A new perfect tetranucleotide microsatellite locus is also reported for the assessment of genetic variability among *U. cordatus* populations.

## Materials and Methods

### Crab samples

From 2006 until 2008 a total of 319 crabs were collected from six different estuaries as depicted in Fig. 1. At each estuary, all crabs were collected at the same site and at the same day. Tissue samples from a segment of the last pereiopod (mero or propod) were removed using a non-lethal method and immediately preserved in 95–100% ethanol in 2.0 mL-cryovials. All samples were then taken to the laboratory and stored at −20 °C until further use.

### DNA extraction and microsatellite genotyping

Total genomic DNA was extracted from the muscle tissue using a phenol/chloroform-isoamyl alcohol (25:24:1, v:v:v) extraction of Sodium Dodecyl Sulfate (SDS)/proteinase-K digested tissue of each individual (Sambrook, Fritsch & Maniatis, 1989). High molecular weight DNA was isolated by ethanol precipitation and visualized by 0.8% agarose gel electrophoresis using Sybr Safe staining (Invitrogen, Carlsbad, CA, USA). Primer pairs UcSSR-01 to UcSSR-08 (GenBank accession no: FJ483820–FJ483827) (Britto et al., 2009) were used to access eight microsatellite loci. PCRs were carried out according to the reference above in 10 μL reactions. An extra tetranucleotide repeat microsatellite locus was included in the analysis as an additional marker, UcSSR-09 (forward primer: 5′-TTTCCTATCTCCATCTCCTTC-3′; reverse primer: 5′-ACATCATCGCAAATTCAGAG-3′). This locus was isolated under the same conditions described by Britto et al. (2009), and in this study, it has followed the same PCR conditions used for the other primers, however using 56 °C as the annealing temperature. PCR products were screened in 6% denaturing polyacrylamide gels and visualized using silver staining following modified protocol described by Benbouza et al. (2006). Product sizes were determined by comparison to a 10 bp DNA ladder (Invitrogen) and scored manually. The individuals were genotyped to obtain baseline allele frequency information.

### Data analysis

#### Quality control of the data

After genotyping, dataset was analyzed in Micro-Checker 2.2.3 (Van Oosterhout et al., 2004) to estimate: (a) alleles outside of the expected range (allele size shifts); (b) presence of null alleles within each locus following Brookfield-1 parameter. The probability of linkage disequilibrium between pairs of loci was determined using Fisher’s exact test in Genepop (Raymond & Rousset, 1995) with 1,000 dememorization steps, 100 batches and 1,000 iterations per batch.

#### Genetic diversity

All nine microsatellite loci were first characterized using all the 319 individuals collected along the Brazilian coastline. The total number of alleles (*A*) and allelic richness per locus (*A_R_*) were estimated by Fstat 2.9.3 (Goudet, 1995). The polymorphic information content (PIC) was also determined using Cervus 3.0 (Kalinowski, Taper & Marshal, 2007). Observed and expected heterozygosities (*H_O_* and *H_E_*) and exact tests for departure from Hardy–Weinberg expectations (*pHWE*) were performed using Genepop (Raymond & Rousset, 1995). *pHWE* was calculated according to the probability test using 1,000 dememorization steps, 100 batches and 1,000 iterations per batch.

#### Population structure

The genetic differentiation among locations was evaluated by Fstat 2.9.3 (Goudet, 1995), which determined the values of *F* (*F*_IT_), θ (*F*_ST_) and *f* (*F*_IS_) according to Weir & Cockerham’s (1984) estimates for the whole sample set. GenAlEx 6.503 (Peakall & Smouse, 2006) was used to determine the influence of the geographical distance in genetic differentiation, calculating the significance of correlations between “linearized pairwise *F*_ST_,” (*F*_ST_/(1−*F*_ST_)) and coastline geographical distances (according to the one-dimension model; geographical distances in km can be found in Supplemental Information 1, “Coastline_Distance”), using Mantel’s test with 1,000 permutations. The same procedure was also performed with Jost’s *D* index (Jost, 2008) estimated between each pair of locality. Probability values of pairwise comparisons were corrected to control Type I error rate using Bonferroni correction, as implemented in “*p.adjust*” function of R software (R Development Core Team, 2017), with significance value of 0.05.

Evidences of panmixia on the covered sampling area were also tested with the algorithm implemented by spatial analysis of shared alleles (SAShA) (Kelly et al., 2010). Observed and expected mean distance distribution of alleles were estimated using Geographical coordinates of the sampling locations in a run with 1,000 permutation.

Patterns of population structure were first tested by AMOVA, using Arlequin 3.5 (Excoffier, Laval & Schneider, 2005), with 1,000 permutations. Samples from different localities were arranged according to their geographical position into BC and NBC groups. BC group was represented by samples from “CAN and CAR,” while NBC group was constituted by samples from “FOR, CAU, CUR and SOU” (see Fig. 1). Thus, *F*_CT_ value was estimated considering BC *vs* NBC groups. Additionally, another a priori configuration was tested arranging samples into three groups: BC *vs.* NBC-1 (FOR and CAU) *vs.* NBC-2 (CUR and SOU).

The same data was also processed in Samova 2.0 software (Dupanloup, Schneider & Excoffier, 2002), which implements an approach to define groups of populations that are maximally differentiated from each other while run AMOVA analysis. The software was set to arrange samples in several configurations, starting with two groups up to five groups. Tests were performed with and without geographical position information of each locality.

The occurrence of genetic structure among samples were also estimated by Structure 2.3.3 (Pritchard et al., 2000), which implements a Bayesian clustering algorithm where a priori information about the origin of samples were not used. Analyses were carried out according to the admixture model considering that there was no linkage disequilibrium between markers. Ten runs with a burn-in of 200,000 and a run length of 700,000 iterations were performed for a number of clusters varying from *K* = 1 to *K* = 8. The most probable number of populations was estimated according to posterior probabilities of each tested *K* as described by Pritchard et al. (2000).

The R package Adegenet (Jombart, 2008) was employed to run DAPC. This method is intended to identify and describe genetic clusters optimizing variance between clusters and minimizing variation within them. Initially the “find.clusters” function were applied to detect the number of clusters that best represents the data starting with a principal components analysis (PCA). The optimal number of clusters (*k*-means) were estimated using different clustering solutions that were compared using Bayesian information criterion (BIC). The groups formed with samples from the different sites were then organized in a table whit rows corresponding to actual groups (“sample sites”), while columns correspond to inferred groups. Afterwards the “dapc” function was used and a new PCA was performed. At this time, the number of components representing at least 85% of the total variance of the data were chosen and the number of eigenvalues to be followed in the discriminant functions were defined. The results were showed graphically in a scatter plot with each point corresponding to a specimen.

#### Gene flow models

Migrate 4.2.14 (Beerli & Felsenstein, 2001) was used to estimate gene flow among sample sites and the directivity of migration events according to different models. These analysis were performed considering *F*_CT_ results (see AMOVA above), where localities belonging to the same group (*P* < 0.05) were pooled together and analyzed as a single population. Afterwards, the following models of migration with their respective assumptions were tested:

1. Directional migration 1: gene flow follows preferentially from North to South Brazilian coast;
2. Directional migration 2: gene flow follows preferentially from South to North Brazilian coast;
3. Directional migration 3: gene flow follows BC (southwards) and NBC (northwards) direction starting from the bifurcation of these main currents;
4. Stepping stone model: there is symmetrical migration among neighbors and no direct migration among non-neighbor localities;
5. Island model: there is a constant gene flow among each sample site;
6. Panmixia: there is a single and huge population with random mate.

The Migrate notation for each model is presented in results section following the number of groups established by AMOVA. Replicates were run using the microsatellite model in a single step method with constant mutation rate and starting parameters based on *F*_ST_ calculations. Prior distribution was set to be uniform for the mutation-scaled effective population size Θ (= 4*N_e_*μ) and mutation-scaled migration rates *M* (= *m*/μ). Runs were replicated with different random seeds four times to ensure convergence. The effective number of migrants (*N_e_m*) was estimated by the relation *N_e_m* = (Θ × *M*)/4. Calculation was performed with a burn-in of 10,000 iterations for each chain and one long chain with 100 increments and 10,000 recorded steps in each chain. Thermodynamic integration (used to find out marginal Likelihoods) was determined with Bezier approximated score after set a static heating scheme with four chains with temperatures 1.00, 1.50, 3.00 and 500,000.00. A matrix with the pairwise linear distance (in km) between each sample sites was used in the analysis, allowing migration rates to be scaled to by mutation rate and by these distances. Each proposed model was run ten times to check the standard deviations in marginal Likelihood and ensure reliability of estimates.

Natural log Bayes factors (LBF) and the model with the best statistical support (higher probability) were calculated as suggested by Kass & Raftery (1995).

## Results

### Genetic diversity

All microsatellite loci were polymorphic across all six localities sampled. The analyses of 319 individuals collected along the Brazilian coast showed that the total number of alleles ranged from five, for locus UcSSR-05, to 27, for locus UcSSR-06, with an average of 12.4 (s.d. = 7.6) alleles per locus (Table 1). The PIC was estimated for evaluating the level of polymorphism of each locus and vary from 0.453 to 0.939 (average of 0.660; s.d. = 0.164). Observed heterozygosity estimates for *U. cordatus* ranged from 0.349 (locus UcSSR-04) to 0.946 (locus UcSSR-06), whereas expected heterozygosity ranged from 0.440 (locus UcSSR-01) to 0.930 (locus UcSSR-06). Overall mean observed heterozygosity for all nine loci (0.660) was lower than the mean expected heterozygosity (0.679). With the exception of locus UcSSR-04, all microsatellites conformed to Hardy–Weinberg expectations. The significant deviation from HWE (*pHWE* < 0.001) was interpreted as an indication of null alleles with an occurrence probability of 20.1% for this locus (Table 1). When this marker was excluded from analysis, the difference between *H_O_* and *H_E_* estimates was considered negligible, averaging 0.698 and 0.694, respectively.

**Table 1:** Characterization of microsatellite loci for *U. cordatus*, amplified from 319 individuals of six localities.

Locus	Repeat motif	Size range (bp)	*A*	*NA*	*PIC*	*H_O_*	*H_E_*	*F*	θ	*f*	*pHWE*
UcSSR-01	CTCGn	115–143	08	0.005	0.453	0.425	0.440	0.024	0.010	0.014	0.937
UcSSR-02	CTGTn	131–167	10	−0.019	0.757	0.766	0.743	−0.024	0.020	−0.044	0.946
UcSSR-03	ACAGn	111–159	07	0.008	0.725	0.712	0.721	0.024	0.009	0.015	0.988
UcSSR-04*	TCTAn	156–172	06	0.201	0.569	0.349	0.563	0.330	0.006	0.327	<0.001
UcSSR-05	TGTAn	161–177	05	0.016	0.509	0.494	0.503	0.020	−0.001	0.021	0.956
UcSSR-06	ATAGn(N)11GATAn	173–277	27	−0.005	0.939	0.946	0.930	−0.006	0.012	0.018	0.981
UcSSR-07	CATAn/ACAGn	175–215	11	−0.007	0.671	0.646	0.656	−0.013	0.006	0.019	0.100
UcSSR-08	ACTn(N)21CTAn	260-326	21	−0.036	0.702	0.722	0.688	−0.039	0.009	−0.049	0.920
UcSSR-09	CTATn	294–358	17	0.015	0.899	0.872	0.869	0.039	0.048	−0.009	0.243
Mean/Multilocus estimate (for all 9 loci)			12.4		0.691	0.660	0.679	0.032	0.015	0.016	
2.5% lower bound confidence interval								−0.011	0.007	0.013	
97.5% upper bound confidence interval								0.104	0.026	0.050	
Mean/Multilocus estimate (all loci in HWE)			13.3		0.707	0.698	0.694	0.003	0.016	−0.014	
2.5% lower bound confidence interval								−0.016	0.007	−0.029	
97.5% upper bound confidence interval								0.021	0.028	0.003	

**Notes:**

*A*, total number of alleles; *NA*, Null Allele occurrence probability; *PIC*, polymorphic information content; *H_O_*, observed heterozygosity; *H_E_*, “within sample” expected heterozygosity; *F*, estimate of Wright’s *F*_IT_; θ, estimate of Wright’s *F*_ST_; *f*, estimate of Wright’s *F*_IS_; *pHWE*, significance level of departure from Hardy–Weinberg equilibrium.

* Locus UcSSR-04 was not used in the population structure analysis.

Multilocus estimate for θ (analogue to Wright’s *F*_ST_) over all nine loci varied little with or without UcSSR-04 (0.015 and 0.016, respectively), showing low level of differentiation among localities. Estimates of endogamy overall samples (*F*) and within localities (*f*) were lower when this locus was excluded from analysis (Table 1), however, in both situations estimates were close to zero (0.032 and 0.003 for *F*; 0.0166 and −0.014 for *f*).

Considering the diversity by locality, the mean number of alleles per locus and allele richness varied little among sites (Table 2). Minimum and maximal mean values of *H_O_* found per locality were 0.61 and 0.73, whereas for *H_E_* values ranged between 0.64 and 0.70 (Table 2).

**Table 2:** General sample information and mean diversity indexes per locus for each locality.

Locality	Sample size	Longitude/Latitude	Collection date	*A* (*±s.d*.)	*A_R_* (*±s.d*.)	*H_O_* (*±s.d*.)	*H_E_* (*±s.d*.)
CAN	91	47°59′03″W 25°04′31″S	2008/Jan.	11.3 (5.6)	10.1 (5.0)	0.73 (0.05)	0.70 (0.05)
CAR	51	39°26′55″W 18°01′57″S	2008/Mar.	10.0 (6.4)	09.5 (6.1)	0.71 (0.07)	0.69 (0.05)
FOR	49	37°46′16″W 04°31′52″S	2006/Nov.	08.0 (4.6)	07.8 (4.5)	0.64 (0.07)	0.64 (0.06)
CAU	44	38°46′30″W 03°35′11″S	2006/Nov.	09.0 (5.5)	08.9 (5.5)	0.64 (0.08)	0.67 (0.06)
CUR	42	47°50′36″W 00°39′23″S	2007/Dec.	09.5 (5.9)	09.5 (5.9)	0.63 (0.07)	0.68 (0.06)
SOU	42	48°33′11″W 00°42′30″S	2007/Dec.	09.4 (5.9)	09.4 (5.9)	0.61 (0.07)	0.65 (0.05)
Mean/locality	53.2 (s.d. = 18.9)	—	—	9.5	9.2	0.66	0.67

**Notes:**

All estimates are followed by their respective standard deviations within parenthesis.

*A*, mean number of allele per *locus*; *A_R_*, mean allelic richness per *locus*; *H_O_*, mean observed heterozigosity per *locus*; *H_E_*, mean expected heterozigosity per *locus*.

Since UcSSR-04 showed significant presence of null alleles, it was removed from population structure and gene flow analyses. The quality of data was also observed considering the independence of these markers and, all pairwise tests of linkage disequilibrium were non-significant when comparing all loci in each population as well as across all populations at the 0.05 level of significance.

### Population structure

Pairwise *F*_ST_ and Jost’s *D* evidenced low genetic differentiation between localities (Mean *F*_ST_ = 0.013; s.d. = 0.004/Mean Jost’s *D* = 0.034; s.d. = 0.021) (Table 3). Mantel test performed with genetic distances and linear geographical coastline distances (Supplemental Information 1, “Coastline_Distance”) showed no significant correlation (Mantel_*FST*_: *R*^2^ = 0.04, *P* = 0.220; Mantel_*Jost′sD*_: *R*^2^ = 0.10, *P* = 0.140).

**Table 3:** Triangular distance matrices (used for computation of Mantel test with 1,000 permutations) among *U. cordatus* sample localities.

	CAN	CAR	FOR	CAU	CUR	SOU
CAN	—	**0.009**	**0.022**	**0.012**	**0.011**	**0.012**
CAR	**0.029**	—	**0.020**	0.011	0.009	**0.014**
FOR	**0.082**	**0.069**	—	0.010	**0.016**	**0.018**
CAU	**0.038**	0.026	0.019	—	0.010	0.011
CUR	**0.030**	0.018	**0.044**	0.019	—	0.005
SOU	**0.034**	**0.037**	**0.053**	0.021	−0.004	—

**Note:**

Above the diagonal are linearized pairwise *F*_ST_. Below the diagonal are linearized pairwise Jost’s *D*. Values in bold represent significant differences (*P* < 0.05) between localities.

Regardless of these findings, pairwise comparisons of the genetic distances showed statistical significant differences among several localities. The southernmost sample (CAN) showed significant genetic differences from all other localities. As well, samples from FOR were statistically different from most other localities, with exception to its neighbor CAU. Neighboring localities CUR and SOU have also not shown significant differences (Table 3). CAR samples, which were under the influence of BC, showed no significant differences from CAU and CUR (samples under the NBC influence; see Fig. 1), however, significant differences were found when it was compared to other samples. Despite of the significant differences between the most distant localities, and the absence of differences between the closest ones, no pattern of differentiation could be observed in general.

The assumption of panmixia events was statistically discarded after running SASha, which has shown a non-panmictic condition (*P* = 0.011), albeit with a small difference between the observed mean distance distribution of alleles (2,055.0 km) when compared with the expected mean distance distribution (2,079.7 km).

AMOVA has shown that only 0.71% of the total genetic variance is explained by differences among sample sites, although the variance was significant (*F*_ST_ = 0.019; *P* < 0.001). Considering the variation between BC and NBC groups, no differences were found (*F*_CT_ = 0.007; *P* = 0.141), as well as for comparisons among BC *vs* NBC-1 *vs* NBC-2 (*F*_CT_ = 0.011; *P* = 0.069). However, Samova results suggested an alternative grouping arrangement with significant differentiation for samples organized in four groups, named hereafter as: **BC-1** (“CAN”); **BC-2** (“CAR”); **NBC-1** (“FOR and CAU”); **NBC-2** (“CUR and SOU”). Following this indication *F*_CT_ value remained low, but was significant (*F*_CT_ = 0.037; *P* = 0.023).

**Figure 2:**
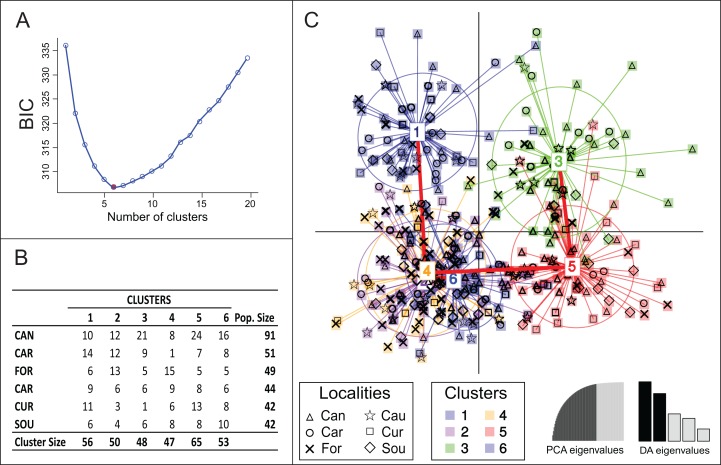
(A) Optimal number of clusters (= 6) defined by the lowest value of BIC (Bayesian Information Content). (B) Matrix of actual groups and inferred clusters. (C) Sample distribution according to discriminant analysis of principal components (red lines connecting numbers 1–6 represent a minimum spanning tree).

Bayesian clustering using Structure suggested lack of genetic differentiation among Brazilian samples with one single genetic group being detected (Posterior probabilities ≈ 100% for *K* = 1). However, another clustering solution suggested by DAPC analysis estimated the occurrence of six genetic groups (Fig. 2). The model was defined after retaining 35 principal components, which explained 85% of the total genetic variance. None of the six groups were completely isolated, with clusters 2, 4 and 6 being totally overlapped; their connectivity was also supported by minimum spanning tree (Fig. 2C), which presented three branches, all connected to Cluster 4. Clusters 1, 3 and 5 were the most divergent with more isolated samples (Fig. 2C). None of the clusters was exclusively represented by a single locality (Figs. 2B and 2C) and no pattern of genetic structure due to geographical position was observed.

### Gene flow inferences

To infer gene flow the four clusters indicated by AMOVA (BC-1, BC-2, NBC-1 and NBC-2) were used. After testing different migration models among these clusters, the most probable one was defined as the island model, with ≈ 100% of probability (Table 4). Other tested models were not statistically supported with probabilities approaching zero. These included Panmixia and the hypothesis of larval dispersal following the main currents (BC and NBC), which were considered the less probable models. Stepping stone and directional migration models were also discarded after analysis.

**Table 4:** Comparison of different migration models for *U. cordatus* sample localities grouped according to *F*_CT_ results.

	Tested models	T.I.	St. dev.	LBF	Model probability	Migrate notation
1	North to South	−7539.2	38.7	−1060.5	0.56 × 10^−230^	{**** 0*** 00** 000*}
2	South to North	−7550.8	18.4	−1083.6	0.49 × 10^−235^	{*000 **00 ***0 ****}
3	Following BC or NBC	−14702.7	789.9	−15387.5	0.00	{**00 0**0 0**0 00**}
4	Stepping stone	−7566.7	8.7	−1115.5	0.59 × 10^−243^	{*s00 s*s0 0s*s 00s*}
5	Island	−7009.0	33.9	0.0	≈1.00	{mmmm mmmm mmmm mmmm}
6	Panmixia	−15717.8	32.4	−17417.7	0.00	{*}

**Note:**

Thermodynamic integration (TI) and log Bayes factor (LBF) were used to estimate the model probabilities. Migrate notation was established according to localities arranged as BC-1, BC-2, NBC-1 and NBC-2, except for the Panmixia model, where all population were pulled together as a single group.

Mean estimate of Θ after ten runs was 0.098 (s.d. = 2.75 × 10^−5^) and mutation-scaled historical migration rates (*M*) were 975.59 (s.d. = 0.974). Considering the island model, which assumes a symmetrical migration rate, the estimated indexes (and *M*) must be considered the same for all pair of population compared. Thus, a single value of effective number of migrants exchanged per locality was defined (23.94 crabs per generation).

## Discussion

*U. cordatus* displays average genetic multilocus diversity with number of alleles, observed heterozygosity and PIC mean values of 12, 0.66 and 0.69, respectively. One caveat needs to be taken into consideration regarding the samples obtained: although the species is present along the Brazilian coast, we could only obtain samples from a few locations. That was main due to limited available survey budget. The present results fill in a gap in knowledge on this species.

According to Botstein et al. (1980), markers with *PIC* values higher than 0.5 are very informative and here, only locus UcSSR-01 presented lower value (*PIC_UcSSR_*_− 01_ = 0.453). Among all loci, only UcSSR-04 was not in Hardy–Weinberg equilibrium (HWE), which may be due to the occurrence of null alleles and therefore, heterozygous individuals would have been scored as homozygous causing HWE deviations (Callen et al., 1993). Nevertheless, overall genotyping data revealed high quality of these microsatellites, and all other markers have passed in quality control. Excluding locus UcSSR-04, differences between *H_O_* and *H_E_* estimates were small, averaging 0.66 and 0.67 respectively. Oliveira-Neto et al. (2014) reported similar values for *U. cordatus* samples from other estuaries with mean values of 0.74 and 0.73 for *H_O_* and *H_E_*, respectively.

Multilocus estimates for Weir and Cockerham’s θ, including or excluding locus UcSSR-04, differed slightly (0.015 *vs* 0.016). In both situations estimates showed low levels of genetic differentiation among localities and no evidence of population structuring. Low levels of inbreeding in the species was detected after *F* and *f* calculations. Linearized pairwise *F*_ST_ and Jost’s *D* have also evidenced weak genetic differentiation among localities, in agreement with θ estimates and with previous results (Oliveira-Neto et al., 2007a, 2007b; Britto et al., 2011). In addition, non-significant results were obtained from Mantel test, supporting the hypothesis of extensive gene flow promoting weak genetic variation among populations, a possible consequence of an efficient larval dispersal strategy of the species. Additionally, Structure also suggested the absence of preferential clusters along the Brazilian coastline, in agreement with previous finding (Oliveira-Neto et al., 2014).

Contrasting with Structure results, the approach based on DAPC analysis suggested the occurrence of six genetic groups, albeit without correlation with sample localities. Janes et al. (2017) advise for problems of many studies over- or underestimating population genetic structure when only Structure results are considered. Jombart & Collins (2015) pointed that the concept of “true *k*” is fairly hypothetical and does not mean that clustering algorithms should necessarily be discarded, but surely the reality is more complex than a few clear-cut, isolated populations. These considerations are important since population dynamics of *U. cordatus* seems to be more complex than it was supposed before DNA molecular data was available. Furthermore, despite of the indication of six groups by DAPC results, each one of them should not be considered an isolated genetic entity, as clear overlaps could be graphically observed among them.

High gene flow between estuaries due to species pelagic larval export strategy is a plausible explanation for our findings. In numerous crab species with similar dispersal strategy larvae are thought to be transported up to 100 km seaward from its spawning location (Epifanio, Masse & Garvine, 1989; Diele, 2000; Epifanio & Garvine, 2001). Our study and the Oliveira-Neto et al. (2014) found statistical differences when southernmost samples were compared with other from Brazilian Northeast and North estuaries. CAN location is situated in São Paulo state, which is located in Southeast Brazil, while southernmost samples from Oliveira-Neto et al. (2014) belongs to Paraná state, which is located in South region of Brazil. Despite of the low values of genetic differences found, southernmost samples were significant different from other localities, suggesting that irrespective of the absence of a clear population structuring pattern, the partitioning of the genetic diversity among Brazilian estuaries is not random distributed. Samova showed significant differences among groups (*P* = 0.023) when localities were arranged as BC-1, BC-2, NBC-1 and NBC-2, despite of the low *F*_CT_ (0.037). SASha results also pointed to a non-panmictic situation for *U. cordatus* crabs, albeit with a small difference between the observed mean distance distribution of alleles (2055.0 km) and the expected mean distance distribution (2079.7 km).

The gene flow models run in Migrate have also not suggested a scenario with completely free exchange of alleles among all sampling localities, and panmixia model was as well discarded. These two current branches run in opposite directions along the Brazilian coast (Fig. 1), and larger genetic differentiation between northern and southern populations might be expected if only the presumed effects of these currents on migration are taken into account. BC flows at about 0.5–0.6 m/s (Evans, Signorini & Miranda, 1983) and NBC at 0.6–1.0 m/s (Arnault et al., 1999), hence organisms living under the influence of these currents (i.e., larval *U. cordatus* specimens) could potentially be transported over several 100 km in only one month. However, there is no evidence to support the main Brazilian currents (BC and NBC) as having a strong barrier effect on larval dispersion.

The model that best described *U. cordatus* migration patterns was the island model. The Bayesian approach of Migrate allows the estimation of gene flow without the assumption of symmetric migration rates or equal population sizes (Beerli & Felsenstein, 2001), nevertheless, our results support symmetric dispersal in this species. We could not identify any location as a potential source or sink of individuals. Results show an average high number of individuals (≈24) per generation in a species with overlapping generations (Alcântara-Filho, 1978; Schmidt & Diele, 2009).

The dispersal of the *U. cordatus* larvae is yet poorly studied in the natural environment. Further studies on oceanographic transport processes and dispersion patterns should elucidate what happens with the planktotrophic larvae when exported through the estuarine plume. If larvae are transported only by ocean surface currents, one would expect a predominantly southward dispersal along the Brazilian Current and northward dispersal along the North Brazilian Current. Another possible explanation is that the larvae are transported by subsurface currents in deeper waters, which could flow in the opposite direction to the surface current (Schott, Fischer & Stramma, 1998). This hypothesis suggests an active migration of larvae under deeper water currents. In fact, *U. cordatus* larvae perform downward migration, but only during the later development stages, when the pelagic-benthic transition is successfully completed and the larvae dive toward the bottom sediment (Silva, 2007). Therefore, during the first zoeal stages, *U. cordatus* larval dispersal is still dependent on surface currents. On the other hand, local wind-driven currents might also explain larval connectivity (Roman & Boicourt, 1999). Although this hypothesis seems more plausible for micro- and meso-tidal regimes, in which estuarine plumes are more susceptible to local wind-driven currents, it is less likely for macro-tidal regimes in which estuarine plumes can reach long distances from the coast. The given possible explanations do not exclude each other, and they might occur simultaneously to sustain high levels of multidirectional larval transport that can explain the genetic connectivity observed in this study.

Is the dispersal rate estimated for *U. cordatus* sufficient to maintain only weak differentiation between locations? According to Wright (1931) statement, only one migrant per generation (the One-Migrant-per-Generation rule—OMPG) would be enough, under certain circumstances, to guarantee low level of genetic differences among populations. This rule can be applied to many conservation and management purposes, however, this concept does not imply panmixia among individuals of different subpopulations. OMPG should be interpreted as a qualitative evaluation of tradeoffs between losses of genetic diversity within populations versus homogenization among populations. The rule results from a simple model of population structure based upon a host of unrealistic assumptions, which could be better applied considering 1–10 migrants per generation (Mills & Allendorf, 1996) and effective number (*Ne*) rather than sample size (*N*) (Wang & Whitlock, 2003). In addition, little information is available on natural selection of *U. cordatus* and its impact on the genetic diversity of the species.

In the absence of extreme selective forces, the homogenizing effects of dispersal, even for small levels of effective migration, can be sufficient to overcome population differentiation (Thorpe, Solé-Cava & Watts, 2000). Nevertheless, despite of the high gene flow among localities, the significant genetic differences among them (according to Jost’s *D*, *F*_ST_ and *F*_CT_) demonstrate that migration rates are actually not counterbalancing the loss of diversity due to genetic drift or selection events. Therefore, the reported levels of exploitation (anthropic selection) may promote a negative impact on populations (Duarte et al., 2014). Under these circumstances, even with high levels of larval dispersal, a more specific conservation policy is necessary to be provided by the Brazilian government in order to maintain healthy crab stocks.

Apart from whether exploitation of crab stocks in Brazil is a sustainable fishing activity, divergence of adult traits can occur due to differential pressure induced by strong fishing efforts. In several species, exploitation concentrated on larger individuals, has been demonstrated to result in constant reduction in age and length of maturity (Haugen & Vøllestad, 2001; Sinclair, Swain & Hanson, 2002). *U. cordatus* populations in Brazil, under uncontrolled fishing pressure, are already assuming a different size profile, in which the average crab size was reduced considerably (Jankowsky, Pires & Nordi, 2006). For example, a survey of the morphological pattern of crabs from Espírito Santo (Brazil) revealed that the mean carapace width of the population was 4.89 cm, below the minimum legal harvesting size (6.13 cm) (Conti & Nalesso, 2010). This type of variation could not be assessed with the present molecular markers. Moreover, because the commercial capture is allowed only for the collection of male individuals, the predominance of females over males at some sites (especially close to villages) brought out the advanced level of overfishing of this population and highlighted the urgent need of management actions (Conti & Nalesso, 2010; Goês et al., 2010).

Thus, even with crab populations presenting homogeneous genetic distribution along the Brazilian coast and with high migration rates, the significant differences found among some localities cannot be rejected. *U. cordatus* populations are not panmictic and despite of the weak population structure, it seems that gene flow does not completely bypass the effects of drift, natural selection (e.g., lethargic crab disease) or human exploitation, which is excessive and should be taken into account for designing species conservation programs. Statistical models suggest that migration events occur following island model, with symmetrical rates of migration among locations. Thus, the connectivity observed between crab populations evidenced the importance of a cooperative work over a wide geographic range aiming at the conservation and management of this fishery resource.

## Supplemental Information

10.7717/peerj.4702/supp-1 Supplemental Information 1 Geographical coastline distances (in Km) between sampled localities.

10.7717/peerj.4702/supp-2 Supplemental Information 2 Raw data. The genotypes (raw data) used in the analyzes performed to calculate the estimates used in the manuscript. The data is distributed in the GENPOP software format (http://genepop.curtin.edu.au).

## Additional Information and Declarations

### DNA Deposition

The following information was supplied regarding the deposition of DNA sequences: GenBank accession numbers: FJ483820–FJ483827, FJ483820.1, FJ483821.1, FJ483822.1, FJ483823.1, FJ483824.1, FJ483825.1, FJ483826.1, FJ483827.1.

### Data Availability

The following information was supplied regarding data availability: The raw data has been supplied as a Supplemental File.
